# Comparison of Integrated Responses to Nonlethal and Lethal Hypothermal Stress in Milkfish (*Chanos chanos*): A Proteomics Study

**DOI:** 10.1371/journal.pone.0163538

**Published:** 2016-09-22

**Authors:** Chia-Hao Chang, Cheng-Hao Tang, Chao-Kai Kang, Wan-Yu Lo, Tsung-Han Lee

**Affiliations:** 1 Department of Life Sciences, National Chung Hsing University, Taichung 402, Taiwan; 2 Department of Oceanography, National Sun Yat-Sen University, Kaohsiung 804, Taiwan; 3 Tainan Hydraulics Laboratory, National Cheng Kung University, Tainan 709, Taiwan; 4 Department of Biotechnology, Hung Kuang University, Taichung 433, Taiwan; 5 Agricultural Biotechnology Center, National Chung Hsing University, Taichung 402, Taiwan; Institut National de la Recherche Agronomique, FRANCE

## Abstract

Milkfish is an important aquaculture species in Taiwan, and its high mortality during cold snaps in winter usually causes huge economic losses. To understand the effect of hypothermal stress and the corresponding compensatory stress response in milkfish, this study aimed to compare liver and gill protein levels between milkfish exposed to nonlethal (18°C), lethal (16°C), and control (28°C) temperatures. Using a proteomics approach based on two-dimensional electrophoresis and nano-LC-MS/MS analysis, this study identified thirty unique protein spots from milkfish livers and gills for which protein abundance was significantly different between nonlethal, lethal, and control temperature groups. Proteins identified in the liver were classified into three different categories according to their cellular function: (1) anti-oxidative stress, (2) apoptotic pathway, and (3) cytoskeleton. Similarly, proteins identified in the gill were sorted in five different functional categories: (1) cytoskeleton, (2) immune response, (3) protein quality control, (4) energy production, and (5) intracellular homeostasis. Based on functional information derived from the identified proteins, we assumed that different levels of hypothermal stress had a different effect and induced a different cellular response. Upon nonlethal hypothermal stress, the identified proteins were involved in anti-oxidative stress and anti-inflammation pathways, suggesting that milkfish had high levels of oxidative stress in the liver and exhibited inflammation response in the gill. Upon lethal hypothermal stress, however, identified proteins were associated with apoptosis in the liver and regulation of intracellular homeostasis in the gill. The present study provided evidence to illustrate different multi-physiological responses to nonlethal and lethal hypothermal stress in milkfish livers and gills.

## Introduction

Over the past decade, global warming has had a great impact on the environment, resulting in increased climate variability and extreme weather conditions. Owing to climate change, cold snaps and ice storms are more likely to occur during winter [1,2]. Most organisms on earth, including fish, are ectotherms, having the ability to survive and adapt to temperature fluctuations [3,4]. Temperature affects all aspects of physiology by influencing processes involving reaction rates as well as physical properties of biological molecules [5]. Based on their thermal tolerance range, ectothermic fishes fall into two distinct species: eurythermal and stenothermal fishes. Eurythermal fishes such as the channel catfish (*Ictalurus punctatus*) have the ability to tolerate a large range of thermal fluctuations (from near freezing point to over 36°C) [6]. In contrast, stenothermal fishes such as the Antarctic icefish (*Trematomus bernacchii*), spend their entire life in the ocean at around -1.8°C and their critical hot temperature is near 6°C [7,8].

Several studies have reported mass mortality of different fish species during winter and overwinter [9–12]. Gilthead sea breams (*Sparus aurata*) are sensitive to low temperature, which leads to body weight loss and higher mortality, a condition referred to as the “Winter syndrome” [10,12,13]. Lethal and nonlethal temperatures were determined for marine teleosts to study their thermal tolerance. Critical thermal tests require relatively few individuals and little equipment and can provide a rapid, nonlethal assessment of thermal tolerance [8,14–17]. Critical temperature minimum (CTMin) and survival temperature minimum (STMin) were estimated for the brown-marbled grouper (*Epinephelus fuscoguttatus*) and the yellowfin seabream (*Acanthopagrus latus*). When acclimated to environments similar to their original habitats, these fishes were more tolerant to cold shock [14,17]. Rapid decrease in water temperatures may affect physiological and behavioral processes in most fish species. Duration and magnitude of the cold shock are significant factors that affect hypothermal stress response in fish [11–13]. Donaldson et al. [11] stated that cold shock stress generally induced a three-stage response in fish. According to the authors, secondary response to cold stress comprises metabolic, cellular, hematological, osmoregulatory, and immunological responses [11].

Exposure to environmental stress such as low temperature might increase oxidative stress levels and induce transition to anaerobic metabolism [18]. The liver of vertebrates is a biochemical factory with multiple physiological functions. Fluctuations in temperature cause rapid release of cortisol, resulting in the stimulation of both gluconeogenesis and lipolysis in the liver [18]. The gill is a specialized organ found in many aquatic organisms that is in direct contact with the external environment. Among other functions, fish gills play an important role in maintaining the homeostasis of internal environments by regulating the physiological response to environmental stresses [19–23]. Up to the present time, proteomics techniques have been implemented in several different studies on fish [24–28]. Among those, a study by Ibarz et al. [24] focused on the impact of cold stress on teleosts, and indicated that exposure to low temperature could lead to oxidative stress in the liver. In addition, several other studies have shown that changes in ambient temperature can have a great impact on ectotherms [29,30]. In the light of these findings, we hypothesized that nonlethal hypothermal stress stimulates the activation of complex physiological stress response mechanisms in vital organs of fish such as gill and liver, while lethal hypothermal stress seriously threatens physiological homeostasis by disturbing the regulation of various cellular pathways.

Milkfish (*Chanos chanos*) is one of the most important aquaculture species in Taiwan. Every year, milkfish die in large numbers during the cold snap in winter [31]. Therefore, a comprehensive comparison of the effects of nonlethal and lethal temperatures on milkfish should be conducted to gain insight into the mechanisms responsible for milkfish mortality due to hypothermal stress. In the present study, nano-LC-MS/MS was used to analyze proteomic profiles of livers and gills from seawater-acclimated milkfish under normal (28°C), nonlethal (18°C), and lethal (16°C) temperatures. Uniquely identified proteins were compared between milkfish exposed to different levels of hypothermal stress and classified into functional categories to elucidate their role in metabolic and cellular responses pathways.

## Materials and Methods

### Ethics statement

The protocol detailing the experiments on fish was reviewed and approved by the Institutional Animal Care and Use Committee (IACUC) of the National Chung Hsing University (IACUC Approval No. 98–110 to THL). Optimal environments for experimental fish were maintained by carefully checking the fish twice per day. 100 L tanks were used in the hypothermal experiments with continuously circulation and filtration. The conditions of swimming and food intake of all fish were monitored every day. All tanks were covered on top to avoid jump-out of the milkfish to minimize unexpected deaths. In addition, the humane endpoints were used during the preliminary studies of milkfish survival tests and the following hypothermal experiments. When milkfish in the hypothermal experiments lost swimming ability or equilibrium, the fish were euthanized in 2.0% 2-phenoxyethanol to the endpoint. All surgery was performed under 0.5% 2-phenoxyethanol anesthesia, and all efforts were made to minimize suffering and distress.

### Experimental animals

Juvenile milkfish (*Chanos chanos*) with average total length of 9.36 ± 0.14 cm and body weight of 10.46 ± 0.51 g were obtained from a local fish farm in Taiwan. Seawater (SW; 35‰) was prepared from the local tap water with proper amounts of RealOcean^™^ Synthetic Sea Salt (Camarillo, CA, USA). The milkfish were reared in SW at 28 ± 1°C with a 12/12 hours (h) light/dark photoperiod for at least four weeks before experiments. The water was continuously circulated through fabric-floss filters, and milkfish were fed commercial pellets daily. Feeding was terminated 24 h prior to the experiments.

### Experimental design for hypothermal treatments

For hypothermal treatment, the milkfish were transferred into tanks equipped with a cooling system (TFC-300B, Tung Fa, Taipei, Taiwan) and the temperature was reduced at a constant rate (2°C/h). In the preliminary test, no mortality was observed when milkfish were exposed to 18°C for 14 days, while three fish died at 5 days post-exposure to 16°C. Ten individuals were used in each group (total 20 milkfish). These three fish in the 16°C group lost swimming ability or equilibrium were euthanized in 2.0% 2-phenoxyethanol to the endpoint. Therefore, treatment temperatures for the nonlethal and lethal hypothermal groups were set at 18°C and 16°C, respectively. The exposure period was 4 days for all groups. For the control group, the milkfish were transferred to a tank with water temperature of 28°C. No mortality was found in all groups. The samples for the following analyses were collected from three individuals of each group (total nine fish). Before sampling, fish were anesthetized with 0.5% 2-phenoxyethanol, then sacrificed by cutting the spinal cord.

### Protein extraction

The fish were sacrificed after 4 days of exposure, the livers and gills were dissected out, and the gill arches were further excised. Livers and gills were frozen in liquid nitrogen immediately and subsequently stored at -80°C. Next, livers and gills were homogenized with 0.5 mL lysis buffer (8 M urea and 4% CHAPS) containing protease inhibitor cocktail (v/v: 25/1, #11836145001, Roche, Indianapolis, IN, USA) using an automated tissue homogenizer (MagNA Lyser; Roche Diagnostics, Penzberg, Germany), and lysates were centrifuged at 8000 rpm for 20 minutes (min). The Protein supernatants were collected and purified by acetone precipitation, and protein pellets were denatured with sample buffer (8 M urea, 4% CHAPS, 65 mM DTE, and 0.5% ampholytes). Final concentrations were measured using Bio-Rad Protein Assay (Bio-Rad, Hercules, CA, USA), based on the Bradford method according to the manufacturer’s instructions.

### Proteomic analysis

Two-dimensional electrophoresis (2-DE) was performed according to Lo et al. [32]. Extracted proteins, 250 μg per sample, were used for 2-DE separation. The rehydration solution containing the sample was then placed into a 17-cm immobilized pH gradient (pH 4–7) IPG strip (ReadyStrip IPG strip; Bio-Rad) and left overnight. First-dimension electrophoresis was carried out at 60 kVh (PROTEAN IEF cell, Bio-Rad). IPG strips were equilibrated with 3 mL of equilibrating solution containing 50 mM Tris-HCl (pH 8.8), 6 M urea, 30% glycerol, 2% SDS, a trace of bromophenol blue, and DTE (1% w/v) for 20 min, followed by a second equilibration for 20 min in the same equilibrating solution containing iodoacetamide (2.5% w/v) instead of DTE. The strips were then placed on top of 12% polyacrylamide gels and held in position with molten 0.5% agarose in running buffer containing 25 mM Tris, 0.192 M glycine, and 0.1% SDS. All gels were run at 16 mA for 30 min, followed by 50 mA for 4–5 h. Gels were stained with silver nitrate and all 2-DE maps were repeated at least three times. All protein spots on the 2-DE gels were quantified and compared using the PDQuest software (version 7.1.1; Bio-Rad). In order to investigate tissue proteins unique to each individual milkfish group, we screened the data for protein spots that were present exclusively in the 16°C, 18°C, or 28°C group and we analyzed those by nano-electrospray mass spectrometry (nano-LC-MS/MS).

### Enzymatic digestion, nano-LC-MS/MS analysis, and database searching

Following the above screening, protein spots (1–2 mm in diameter) that were present exclusively in the 16°C, 18°C, or 28°C group were excised from the gels with a pipette tip and transferred into a microcentrifuge tube (0.6 mL). The gel pieces were washed twice with 50 μL of 50% acetonitrile (ACN): 50% 200 mM ammonium bicarbonate for 5 min and shrunk with 100% acetonitrile until they turned white. The gel pieces were then dried for 5 min in a speed vac. The gel pieces were rehydrated at room temperature in 15 μL of 50 mM ammonium bicarbonate at 37°C for 4 min. An equivalent volume of trypsin (Promega, Madison, WI, USA) solution (20 ng/μL) in 50 mM ammonium bicarbonate was then added and the gel pieces were incubated at 37°C for 4 h. After digestion, the gel pieces were vortexed and spun down, and supernatant peptide solutions were stored at -80°C until mass spectrometry analysis.

All analyses were performed using an Ultimate capillary LC system (LC Packings, Amsterdam, The Netherlands) coupled to a QSTARXL quadrupole-time of flight (Q-TOF) mass spectrometer (Applied Biosystem/MDS Sciex, Foster City, CA, USA). Nanoscale capillary LC separation was performed in an RP C18 column. The product ion spectra generated by nanoscale capillary LC-MS/MS (nano-LC-MS/MS) were searched against NCBI databases for exact matches using the ProID program (Applied Biosystem/MDS Sciex) and the MASCOT search program (http://www.matrixscience.com). No taxonomy restriction was used and the mass tolerance of both precursor and fragment ions was set at ± 0.3 Da [33]. The functional categories of all protein spots were identified according to the KEGG database (http://www.genome.jp/kegg/).

### Quantitative analysis of gel images and statistical analysis

Gel images of three replicates per sample were scanned by GS-800 imaging densitometry. 2-DE protein spots were normalized by the total volume of all matched spots, and quantified using the Image J software (version 1.48i, NIH, USA). One-way analysis of variance (ANOVA) followed by Tukey's pairwise test was used to select protein spots significantly different among the three temperature groups.

## Results

### 2-DE analysis of milkfish liver and gill proteins

Protein fractions extracted from the liver and gill of milkfish exposed to lethal low-temperature (16°C), nonlethal low-temperature (18°C), and control temperature (28°C) were separated by 2-DE and stained with silver nitrate. The molecular weight of detected proteins ranged from 14 to 120 kDa, and the isoelectric points ranged from 4 to 7. The number of protein spots detected across all gels for liver and gill fractions were 675–895 and 570–705, respectively. Fully-automated detection and quantification of protein spots were performed using the PDQuest software.

**Fig 1:**
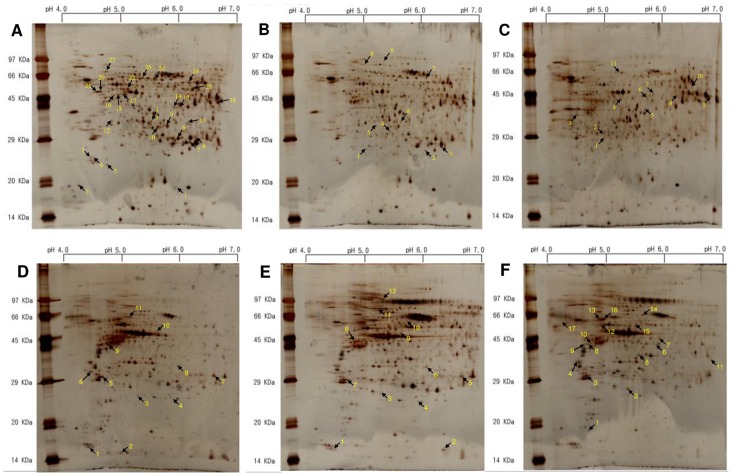
Representative images of silver nitrate-stained 2-DE gels showing liver and gill protein spots corresponding to lethal (16°C; A, D), nonlethal (18°C; B, E) low-temperature, and normal (28°C; C, F) groups. The total protein extraction yields for the same tissue did not differ significantly among the three groups. Fully automated spot detection and quantification were performed using PDQuest software, followed by automated image-to-image matching and statistical analysis. Distinct spots that were identified exclusively in the lethal, nonlethal, or control group were assigned a number for nano-LC-MS/MS analysis.

For both liver and gill protein fractions, 2-DE gel images representative of three replicates experiments per different milkfish group are presented in Fig 1. For the liver fraction (Fig 1A–1C), a total of 27, 9, and 11 protein spots were identified uniquely in the 16°C (Fig 1A), 18°C (Fig 1B), and 28°C (Fig 1C) groups, respectively. For the gill fraction (Fig 1D–1F), a total of 11, 12, 17 protein spots were identified uniquely in the 16°C (Fig 1D), 18°C (Fig 1E), and 28°C (Fig 1F) groups, respectively.

### Protein identification from uniquely identified protein spots

Uniquely identified protein spots were chosen for nano-LC-MS/MS analysis followed by protein identification by MASCOT database search in combination with BLAST search against NCBI database. Proteins that could be identified from 2-DE protein spots obtained from liver and gill protein fractions corresponding to the control, lethal and nonlethal hypothermal groups are listed in Tables 1 and 2. The information listed in Tables 1 and 2 includes protein spot number, accession number, full name and short name of the protein, experimental and theoretical molecular weight and isoelectric point, sequence coverage, and the matching species.

**Table 1:** Comparisons of identified up- and down-regulated proteins in the livers and gills of milkfish between normal (28°C) and nonlethal low-temperature (18°C) environments.

Spot No.^a^	Accession No.^b^	Protein name	Experimental MW(kDa)/pI^c^	Theoretical MW(kDa)/pI^d^	Sequence coverage (%)^e^	Species
**Liver**						
**Down-regulation**						
28–2	Q64374	Regucalcin (Rgn)	31.12/5.01	33.41/5.16	30.8	*M*. *musculus*
**Up-regulation**						
18–3	O08709	Peroxiredoxin 6 (Prdx6)	27.95/6.35	24.87/5.71	23.5	*M*. *musculus*
18–7	Q9NZM5	Glioma tumor suppressor candidate region gene 2 protein (GLTSCR2)	62.73/6.09	54.39/10.32	20.2	*H*. *sapiens*
**Gill**						
**Down-regulation**						
28–2	P47756	F-actin-capping protein subunit beta (CapZ beta)	27.12/5.36	31.35/5.36	20.4	*H*. *sapiens*
28–3	P02760	Protein AMBP (AMBP)	30.31/4.61	39.00/5.95	17.2	*H*. *sapiens*
28–11	P45376	Aldose reductase (AR)	38.57/6.73	35.73/6.71	26.1	*M*. *musculus*
28–12	P01011	Alpha-1-antichymotrypsin (ACT)	62.64/4.86	47.65/5.33	29.0	*H*. *sapiens*
28–13	P11021	78 kDa glucose-regulated protein (GRP78)	72.69/4.83	72.33/5.07	18.9	*H*. *sapiens*
28–16	P11021	78 kDa glucose-regulated protein (GRP78)	73.90/4.90	72.33/5.07	32.3	*H*. *sapiens*
28–17	P50140	Chaperonin homolog Hsp-60, mitochondrial (HSP60)	65.44/4.25	60.10/5.31	30.0	*C*. *elegans*
**Up-regulation**						
18–7	Q96C19	EF-hand domain-containing protein D2 (EFHD2)	29.42/4.71	26.70/5.15	35.7	*H*. *sapiens*
18–11	P0A6M8	Elongation factor G (EF-G)	77.39/5.25	77.58/5.24	25.5	*E*. *coli*
18–12	P50475	Alanine-tRNA ligase, cytoplasmic (AlaRS)	94.86/5.32	106.79/5.41	29.0	*R*. *norvegicus*

^a^. Spot No, spot number in 16, 18 and 28°C 2-DE spot maps.

^b^. Accession No, accession number from NCBI protein database.

^c^. Molecular mass/isoelectric point of experimental protein.

^d^. Molecular mass/isoelectric point of theoretical protein.

^e^. Number of query matched peptides.

**Table 2:** Comparisons of identified up- and down-regulated proteins in the livers and gills of milkfish between lethal (16°C) and nonlethal low-temperature (18°C) environments.

Spot No.^a^	Accession No.^b^	Protein name	Experimental MW(kDa)/pI^c^	Theoretical MW(kDa)/pI^d^	Sequence coverage (%)^e^	Species
**Liver**						
**Down-regulation**						
18–3	O08709	Peroxiredoxin 6 (Prdx6)	27.95/6.35	24.87/5.71	23.5	*M*. *musculus*
18–7	Q9NZM5	Glioma tumor suppressor candidate region gene 2 protein (GLTSCR2)	62.73/6.09	54.39/10.32	20.2	*H*. *sapiens*
**Up-regulation**						
16–1	P14602	Heat shock protein beta-1 (Hspb1)	18.88/5.95	33.83/5.38	19.6	*M*. *musculus*
16–2	P02790	Hemopexin (Hpx)	19.22/4.28	51.68/6.55	34.7	*H*. *sapiens*
16–6	P14602	Heat shock protein beta-1 (Hspb1)	29.69/5.99	33.83/5.38	27.3	*M*. *musculus*
16–7	O08709	Peroxiredoxin 6 (Prdx6)	28.54/6.19	24.87/5.71	23.0	*M*. *musculus*
16–9	P46108	Adapter molecule crk (p38)	34.49/5.81	33.83/5.38	30.5	*H*. *sapiens*
16–10	P07195	Lactate dehydrogenase B chain (LDH-B)	34.03/5.64	36.64/5.71	21.3	*H*. *sapiens*
16–16	Q13233	Mitogen-activated protein kinase kinase kinase 1 (MEKK1)	41.68/6.68	164.47/7.93	35.6	*H*. *sapiens*
16–21	P14211	Calreticulin (CRT)	51.34/4.60	47.99/4.33	27.2	*M*. *musculus*
16–22	P10809	Heat shock 60 kDa protein 1 (Hspd1)	53.57/5.12	61.05/5.70	25.0	*H*. *sapiens*
16–25	P07724	Albumin (Alb)	62.13/5.61	68.69/5.75	17.5	*M*. *musculus*
16–26	P07724	Albumin (Alb)	63.51/5.33	68.69/5.75	26.2	*M*. *musculus*
16–27	P68370	Tubulin, alpha 1a (Tuba1a)	73.75/4.75	50.14/4.94	37.9	*R*. *norvegicus*
**Gill**						
**Down-regulation**						
18–7	Q96C19	EF-hand domain-containing protein D2 (EFHD2)	29.42/4.71	26.70/5.15	35.7	*H*. *sapiens*
18–11	P0A6M8	Elongation factor G (EF-G)	77.39/5.25	77.58/5.24	25.5	*E*. *coli*
18–12	P50475	Alanine-tRNA ligase, cytoplasmic (AlaRS)	94.86/5.32	106.79/5.41	29.0	*R*. *norvegicus*
**Up-regulation**						
16–1	P62158	Calmodulin (CaM)	17.31/4.48	16.83/4.09	28.0	*H*. *sapiens*
16–3	P24534	Elongation factor 1-beta (EF1-beta)	25.58/5.30	24.76/4.50	36.1	*H*. *sapiens*
16–7	P16015	Carbonic anhydrase 3 (CAIII)	30.38/6.64	29.37/6.89	35.7	*M*. *musculus*
16–9	P06576	ATP synthase subunit beta, mitochondrial (ATP5B)	42.33/4.79	56.56/5.26	23.5	*H*. *sapiens*
16–11	P38646	Stress-70 protein, mitochondrial (GRP75)	64.00/5.16	73.68/5.87	30.6	*H*. *sapiens*

^a^. Spot No, spot number in 16, 18 and 28°C 2-DE spot maps.

^b^. Accession No, accession number from NCBI protein database.

^c^. Molecular mass/isoelectric point of experimental protein.

^d^. Molecular mass/isoelectric point of theoretical protein.

^e^. Number of query matched peptides.

For liver samples, a total of 1, 2, and 12 spots contained proteins that were identified exclusively in the control, lethal and nonlethal hypothermal groups, respectively (Tables 1 and 2). For gill samples, a total of 7, 3, and 5 protein spots contained proteins that were identified exclusively in the control, lethal, and nonlethal hypothermal groups, respectively (Tables 1 and 2). In the liver fraction, protein sequences matched mouse (*Mus musculus*, 8 spots), human (*Homo sapiens*, 6 spots), and rat (*Rattus norvegicus*, 1 spot) proteins. In the gill fraction, protein sequences matched human (*Homo sapiens*, 10 spots), mouse (*Mus musculus*, 2 spots), rat (*Rattus norvegicus*, 1 spot), bacteria (*Escherichia coli*, 1 spot), and nematode (*Caenorhabditis elegans*, 1 spot) proteins.

**Fig 2:**
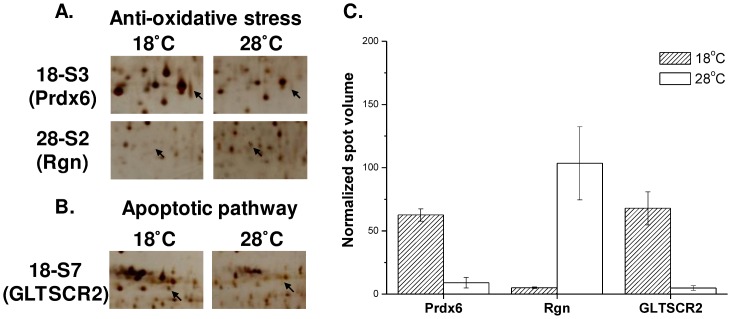
Abundance of proteins identified from livers of the 18°C group compared with that of the 28°C group. The three detected protein spots were categorized into two different functional groups: (A) anti-oxidative stress (i.e., Prdx6, Rgn) and (B) apoptotic pathway (i.e., GLTSCR2). (C) Mean normalized spot volume of liver proteins compared between the 18°C and 28°C groups (n = 3). GLTSCR2, glioma tumor suppressor candidate region gene 2; Prdx6, peroxiredoxin 6; Rgn, regucalcin.

**Fig 3:**
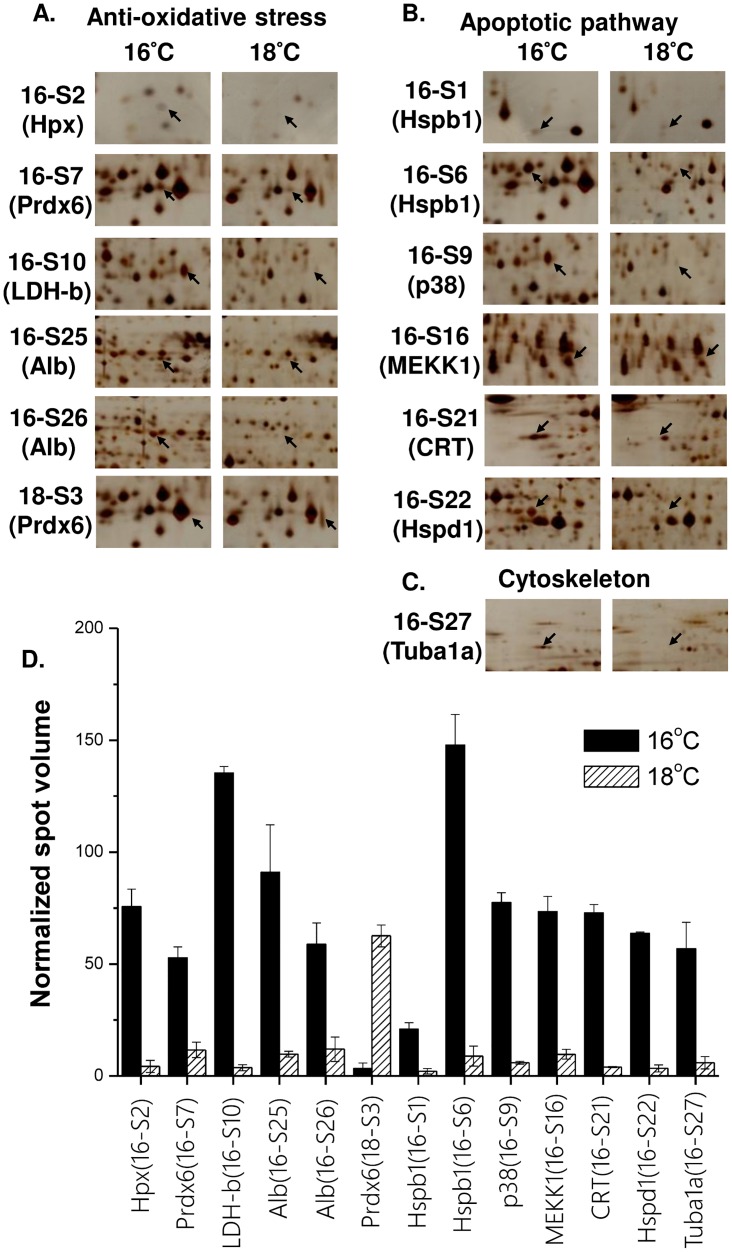
Abundance of proteins identified from livers of the 16°C group compared with that of the 18°C group. Detected protein spots were categorized into different physiological groups: (A) anti-oxidative stress (i.e., Hpx, Prdx6, LDH-b, Alb), (B) apoptotic pathway (i.e., Hspb1, p38, MEKK1, CRT, HSP60), and (C) cytoskeleton (i.e., Tuba1a). (D) Mean normalized spot volume of liver proteins compared between the 16°C and 18°C groups (n = 3). Alb, albumin; CRT, calreticulin; Hpx: hemopexin; Hspb1, heat shock protein beta-1; Hspd1, 60-kDa heat shock protein 1; LDH-b, lactate dehydrogenase B chain; MEKK1, mitogen-activated protein kinase kinase kinase 1; p38, adaptor molecule crk; Prdx6, peroxiredoxin 6; Tuba1a, tubulin, alpha 1a.

**Fig 4:**
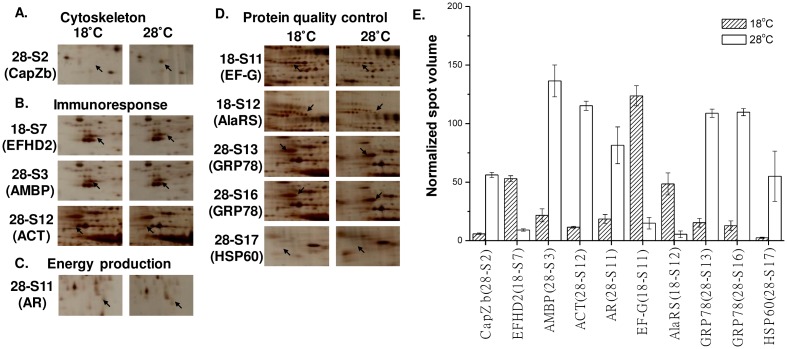
Abundance of proteins identified from the gills of the 18°C group compared with that of the 28°C group. Detected protein spots were categorized into different functional groups: (A) cytoskeleton (CapZb), (B) immunoresponse (EFHD2, AMBP, and ACT), (C) energy production (AR), and (D) protein quality control (EF-G, AlaRS, GRP78, HSP60). (E) Mean normalized spot volume of gill proteins compared between the 18°C and 28°C groups (n = 3). ACT: alpha-1-antichymotrypsin; AlaRS: alanine-tRNA ligase, cytoplasmic; AMBP: protein AMBP; AR: aldose reductase; CapZb F-actin-capping protein subunit beta; EFDH2, EF-hand domain-containing protein D2; EF-G, elongation factor G; GRP78, 78-kDa glucose-regulated protein; HSP60, chaperonin homolog hsp-60, mitochondria.

Four of the identified proteins were detected in more than one different 2-DE spot. These proteins were peroxiredoxin-6 (Prdx6; 18-S3 in Fig 2A and 16-S7 in Fig 3A), heat shock protein beta-1 (Hspb1; 16-S1 and 16-S6 in Fig 3B), albumin (Alb; 16-S25 and 16-S26 in Fig 3A), and 78-kDa glucose-regulated protein (GRP78; 28-S13 and 28-S16 in Fig 4D). Prdx6, Alb, and GRP78 were separated in the horizontal direction of the 2-DE gel, while Hspb1 was separated in the vertical direction of the 2-DE gel.

### Grouping identified proteins by putative function

**Fig 5:**
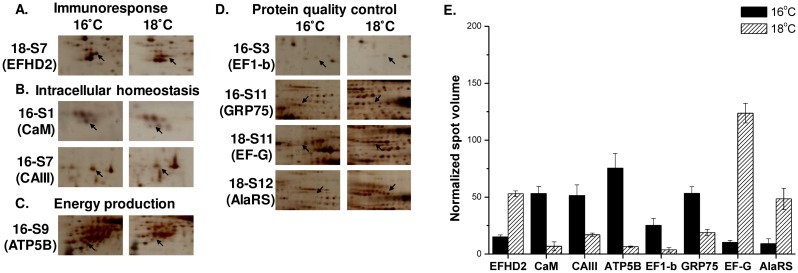
Abundance of proteins identified from the gills of the 16°C group compared with that of the 18°C group. Detected protein spots were categorized into different functional groups: (A) immunoresponse (EFHD2), (B) intracellular homeostasis (CaM, CAIII), (C) energy production (ATP5B), and (D) protein quality control (EF1-b, GRP75, EF-G, and AlaRS). (E) Mean normalized spot volume of gill proteins compared between the 16°C and 18°C groups (n = 3). AlaRS, alanine-tRNA ligase, cytoplasm; ATP5B, ATP synthase subunit beta, mitochondria; CaIII, carbonic anhydrase 3; CaM, calmodulin; EF1-b, elongation factor 1-beta; EFHD2, EF-hand domain-containing protein D2; EF-G, elongation factor G; GRP75, stress-70 protein, mitochondria.

The identified proteins were grouped into different categories based on their functions or metabolic pathway. For the liver fraction, all identified proteins were classified into three categories: (1) anti-oxidative stress, which included lactate dehydrogenase B chain (Ldhb; 16-S10; Fig 3A), hemopexin (Hpx; 16-S2; Fig 3A), albumin (Alb; 16-S25 and 16-S26; Fig 3A), peroxiredoxin 6 (Prdx6; 16-S7 and 18-S3; Figs 2A and 3A), and regucalcin (Rgn; 28-S2; Fig 2A); (2) apoptotic pathway, which included glioma tumor suppressor candidate region gene 2 protein (GLTSCR2; 18-S7; Fig 2B), heat shock protein beta-1 (Hspb1; 16-S1 and 16-S6; Fig 3B), 60-kDa heat shock protein 1 (Hspd1; 16-S22; Fig 3B), calreticulin (CRT; 16-S21; Fig 3B), adapter molecular crk (p38; 16-S9; Fig 3B), and mitogen-activated protein kinase kinase kinase 1 (MEKK1; 16-S16; Fig 3B); (3) cytoskeleton, which included tubulin alpha 1a (Tuba1a; 16-S27; Fig 3C). In the gill fraction, all protein spots were classified into five categories: (1) cytoskeleton, which included F-actin-capping protein subunit beta (CapZ beta; 28-S2; Fig 4A); (2) immune response, which included EF-hand domain-containing protein D2 (EFHD2; 18-S7; Fig 4B), protein alpha-1-microglobulin/bikunin precursor (AMBP; 28-S3; Fig 4B), and alpha-1-antichymotrypsin (ACT; 28-S12; Fig 4B); (3) protein quality control, which included elongation factor 1-beta (EF1-beta; 16-S3; Fig 5D), elongation factor G (EF-G; 18-S11; Fig 4D), cytoplasmic alanine-tRNA ligase (AlaRS; 18-S12; Fig 4D), 78-kDa glucose-regulated protein (GRP78; 28S-13 and 28-S16; Fig 4D), mitochondrial chaperonin homolog hsp-60, (HSP60; 28-S17; Fig 4D), and mitochondrial stress-70 protein (GRP75; 16-S11; Fig 5D); (4) energy production, which included aldose reductase (AR; 28-S11; Fig 4C), and mitochondrial ATP synthase subunit beta (ATP5B; 16-S9; Fig 5C); (5) regulation of intracellular homeostasis, which included calmodulin (CaM; 16-S1; Fig 5B), and carbonic anhydrase-3 (CAIII; 16-S7; Fig 5B).

### Branchial and hepatic protein levels in lethal and nonlethal hypothermal groups

In the liver fraction, Rgn (anti-oxidative stress) was the only protein showing decreased abundance upon hypothermal stress, with protein levels decreasing 20.7-fold upon nonlethal hypothermal treatment compared to that in untreated controls (Fig 2C). Nonlethal hypothermal stress triggered increase in abundance for two identified proteins: GLTSCR2 (apoptotic pathway) and Prdx6 (anti-oxidative stress). Protein levels of GLTSCR2 and Prdx6 increased 8.2- and 14.0-fold, respectively, compared to that in the untreated controls (Fig 2C). Lethal hypothermal stress caused increase in abundance for eight identified proteins: Hspb1, p38, MEKK1 (apoptotic pathway), Ldhb, Hpx, Alb, Prdx6 (anti-oxidative stress), and Tuba1a (cytoskeleton). Among those, HPX, Ldhb, and Hspd1 showed the highest increase, with their protein abundance increasing 97.0-, 26.8- and 16.2-fold, respectively, compared to that in the untreated controls (Fig 3D).

In the gill fraction, six identified proteins, CapZ beta (cytoskeleton), AR (energy production), GRP78, HSP60 (protein quality control), AMBP, and ACT (immune response), showed decreased abundance upon nonlethal hypothermal stress compared to that in untreated controls. Notably, compared to that in the untreated control group, the abundance of CapZ beta (cytoskeleton), AR (energy production), GRP78, HSP60 (protein quality control), AMBP, and ACT (immune response) decreased in the nonlethal hypothermal group by 4.5-, 4.0-, 5.6-, 13.6-, 6.2-, and 5.3-fold, respectively (Fig 4E). Nonlethal hypothermal stress led to increased levels of three identified proteins: EF-G, AlaRS (protein quality control), and EFHD2 (immune response). Protein levels of EF-G, AlaRS, and EFHD2 increased 8.3-, 5.3-, and 3.5-fold, respectively, compared to that in the control group (Fig 4E). Exposure to lethal hypothermal stress resulted in increased abundance for five identified proteins, i.e., EF1-beta and GRP75 (protein quality control), CaM, and CAIII (regulation of intracellular homeostasis), ATP5B (energy production). Compared to that in the control group, the protein abundance of EF1-beta, GRP75, CaM, CAIII, and ATP5B increased 6.3-, 3.5-, 30.6-, 4.0-, and 6.5-fold, respectively (Fig 5E).

## Discussion

In the present study, proteomics analysis was used to identify and quantify liver and gill proteins of milkfish exposed to lethal and nonlethal hypothermal stress. While previous studies have reported the effect of hypothermal stress on other fish species such as tilapia [34], zebrafish [35], and gilthead sea bream [24], this represents the first attempt to compare the effects of lethal (16°C) and nonlethal (18°C) low-temperatures on milkfish. We have identified essential proteins that changed in abundance upon exposure to lethal and nonlethal low-temperatures, and then evaluated compensatory metabolic and cellular responses pathways critical for milkfish survival to hypothermal stress.

### Anti-oxidative stress response in the liver

Four anti-oxidative stress proteins were detected by the proteomics analysis in milkfish liver: lactate dehydrogenase b (Ldhb), hemopexin (Hpx), regucalcin (Rgn), and peroxiredoxin 6 (Prdx6). Ldhb catalyzes the reversible conversion of lactate to pyruvate to produce ATP in the anaerobic phase of the glycolysis. Glycolysis and gluconeogenesis are upregulated in the liver in hypoxic environments and Ldhb is a hypoxia-response marker gene in killifish and Japanese medaka [36,37]. Elevated LDH protein abundance and activity have been reported in the Antarctic mesopelagic fish with increasing environmental temperatures [38]. In our study, increased levels of Ldhb upon lethal hypothermal stress indicated that low-temperatures might have generated an oxygen-limited environment in the liver of milkfish, thereby leading to anaerobic glycolysis in order to generate ATP and thus maintain liver function. Hpx is a heme-binding protein and associated albumin cotransporter involved in heme scavenging, turnover of heme proteins, and protection of free heme from oxidative damage. In addition, Hpx functions include iron homeostasis, antioxidant protection, bacterial infection, and promotion of cell survival [39]. Moreover, an ortholog gene of the mammalian Hpx, known as the warm temperature acclimation associated protein 65-kDa protein (Wap65), has been identified in teleosts. Two paralogs of Wap65, Wap65-1 and Wap65-2, are highly similar in structure to Hpx [40]. Both mRNA and protein levels of Wap65 were found significantly increased upon abnormal temperature environments, and iron has been identified as the pivotal element during bacterial infection and hemolysis in ayu, carp, and goldfish [41–43]. Our proteomics analysis detected an increase in the protein levels of Hpx (Wap65) when milkfish were exposed to lethal low temperature. This suggests that lethal low-temperatures induced an increase in immune response and hemolysis in milkfish liver, and therefore Hpx could be a promising biomarker of lethal hypothermal stress in milkfish.

Rgn plays important roles in calcium homeostasis, protein signal transduction, and vitamin C biosynthesis in the liver [44]. In SMP30/Rgn knockout mice, Rgn was found to have gluconolactonase activity. The lactonase reaction with L-gulono-gamma-lactone is the penultimate step in vitamin C biosynthesis [44]. SMP30/Rgn has been shown to have antioxidant enzyme activity and participates in the suppression of oxidative stress in the liver [45]. Reduced levels of SMP30/Rgn were found to be related to aging in mammals and zebrafish, suggesting that this protein may be linked to signal transduction and regulation of antioxidant mechanisms [46]. In our study, decreased levels of Rgn were detected in the milkfish liver upon nonlethal cold stress compared to that in control groups, implicating that cold temperatures might negatively affect antioxidant activity and increase oxidative stress.

In mammals and teleosts, the peroxiredoxin (Prdx) protein family includes antioxidant enzymes involved in the regulation of signal transduction in the cytosol. Among those, Prdx6 belongs to the class of 1-Cys peroxiredoxins as it contains only one active site Cys residue [47]. Prdx6 was also reported to have glutathione peroxidase activity and phospholipase A2 activity [48]. LPS and poly I:C were used to elucidate specific immune responses and other associated functions of Prdx6 in the yellow croaker [49], the rock bream [50], and the gilthead sea bream [51]. In the rock bream, recombinant Prdx6 has been shown to be involved in maintaining proteins in a reduced state as well as in scavenging harmful ROS and fight toxic marine viruses and bacteria [50]. In our study, a pI shift was detected for two Prdx6 protein spots upon lethal and nonlethal hypothermal stress. A similar pI shift for Prdx6 was previously attributed to different Prdx6 oxidation states in mice [52]. Exposure of milkfish to lethal low-temperature resulted in the detection of a more acidic Prdx6, suggesting that Prdx6 had been oxidized by ROS, while the more basic Prdx6 corresponds to the reduced form of the protein. Therefore, different oxidative states may have led to changes in Prdx6 pI upon exposure to 18°C and 16°C.

### Apoptotic pathway in the liver

Detected liver proteins that were grouped in the anti-apoptotic response pathway included GLTSCR2, Hspb1, Hspd1, CRT, p38, and MEKK1. GLTSCR2 is a novel protein that can bind PTEN or p53 in the apoptotic pathway and functions as a tumor suppressor [53]. Hspd1 and Hspb1, two proteins uniquely identified by 2D-PAGE upon exposure of milkfish to lethal hypothermal environment, might play a critical role in decreasing intrinsic apoptotic effects and liver fibrosis. Hspd1, mainly located in the mitochondria, is involved in the regulation of protein folding and degradation, and it may function as a signaling molecule in the innate immune system. In addition, Hspd1 might interact with caspase-3 in oxidative stress environments [54]. Hspb1 was also reported to block the intrinsic apoptotic pathway by binding p53, Akt, cytochrome c, and caspase-3 [55]. Hspb1 is ubiquitously expressed at low levels in normal cells, but it accumulates in large amounts upon heat shock, cold shock, or oxidative stress, thereby preventing apoptosis by blocking several steps of the apoptotic pathway [56]. In addition, 27-kDa-phosphorylated and 20-kDa-MMP9 cleaved Hspb1forms have been shown to have anti-apoptotic effect and to inhibit VEGF-induced angiogenesis in mammalian cells [57]. Another important protein that was detected in increased abundance upon lethal hypothermal stress is CRT, an endoplasmic reticulum (ER) luminal resident protein, which has lectin-like chaperone properties, and is involved in Ca^2+^ homeostasis and apoptosis inhibition [58]. In the tiger shrimp, it has been shown that CRT is strongly expressed in hemocytes after 1 h of hot temperature stress, and that this protein has the ability to form a complex with reticulum protein 57 [58]. Moreover, the CRT gene was found highly expressed in the liver of the Asian seabass. The CRT gene levels decreased 8-fold in Asian seabass upon exposure for 1–3 h to hypothermal stress before rising back to normal values, suggesting that the CRT gene might have a role in freezing tolerance [59]. The increased levels of CRT protein detected in this study might indicate potential ER stress caused by lethal low-temperature environment. These results will be helpful to understand the mechanisms of physiological response to hypothermal environments in milkfish liver.

### Cytoskeleton in the liver and gill

Most processes of cellular stress response are usually accompanied by cytoskeleton reorganization. Among the milkfish gill proteins that were found decreased in abundance upon cold stress, in comparison with the control group, was F-actin capping protein subunit beta (Capzb). Capzb binds in a calcium-independent manner to the fast growing ends of actin filaments, thereby blocking the exchange of actin subunits at these ends [60,61]. Inhibition of this capping protein may cause shortening of actin filaments leading to pliability changes in the cytoskeletal meshwork and increase in permeability in the gills of milkfish under cold stress. On the other hand, tubulin alpha 1a was increased in the liver of milkfish exposed to lethal hypothermal environment compared to that in the nonlethal group. In eukaryotic cells, tubulin has been found overexpressed under oxidative stress. ROS form GSH-tubulin mixed disulfides, inhibiting actin cytoskeleton remodeling after stress, which is a key process in the regulation of apoptosis and aging [24,62,63]. The results of our study showed that the oxidative stress environment generated upon exposure to lethal cold temperatures might lead to apoptosis in the liver of milkfish.

### Immune response in the gill

Three protein spots were identified as immune response markers in milkfish gill: EFHD2, alpha-1-antichymotrypsin (ACT), and AMBP. EFHD2 is a calcium-binding adaptor protein with two predicted EF-hands. One of its functions is to control the amplitude of the B cell receptor-elicited Ca^2+^ flux from the ER, leading to B cell activation. EFHD2 also terminates this process through a Ca^2+^-mediated feedback inhibition [64]. AMBP is proteolytically processed into two distinct functional proteins, (i) alpha-1-microglobulin, which may play a role in the regulation of inflammatory processes, and (ii) bikunin, which inhibits trypsin and lysosomal granulocytic elastase, and plays an important role in many physiological processes in mammals, e.g. modulating cell growth, blocking cellular calcium uptake, and participating in anti-inflammatory response [65]. ACT, a member of the serine proteinase inhibitors family, inhibits neutrophil proteinase cathepsin G and mast cell chymases [66]. Our results showed decreased levels of AMBP and ACT upon cold stress, indicating that the milkfish might have been exposed to inflammation due to bacterial infection in the gills, and increased abundance of EFHD2, suggesting B cell activation as a response mechanism to promote acclimation to the nonlethal hypothermal environment.

### Protein quality control in the gill

Several proteins identified in gill were associated with protein quality control: elongation factor 1-beta (EF1-beta), elongation factor G (EF-G), alanine-tRNA ligase, cytoplasmic (AlaRS), 78-kDa glucose-regulated protein (GRP78), chaperonin homolog Hsp-60, mitochondrial (HSP60), and stress-70 protein, mitochondrial (GRP75). EF-G is involved in the elongation step of the protein synthesis process. In hypoxic stress conditions, elongation factor G has been found up-regulated after 25 days in zebrafish gill [67], while down-regulation of ubiquitin and up-regulation of translational processes were observed after 6 days in medaka gill [68]. In winter flounder liver, the expression of alanine acyl-tRNA (AlaRS) increased approximately 40% in winter compared to that in summer [69]. In low-temperature environments, it has been shown that large amounts of alanine-rich antifreeze proteins are exported into the circulatory system to satisfy the heavy demand for alanine required for the protein synthesis during cold acclimation [69]. Proteins of the heat shock protein (HSP) family are usually up-regulated in response to stress, and play a role in the regulation of several critical cellular processes involved in protein folding [70]. GRP75, a member of the HSP family, is a protein of mitochondrial origin, but is also found in the ER as well as in the cytosol and in cytoplasmic vesicles. The up-regulation of GRP75 is triggered by glucose deprivation and oxidative injury [71]. In contrast, GRP75-deficient mutant zebrafish exhibit anemia, dysplasia, and cell apoptosis [71]. In the gilthead sea bream, GRP75 is part of a stress response pathway that might be critical for protection against oxidative damage [72]. Glucose regulated protein 78 (GRP78), a member of the Hsp70 family, is exported from the ER and plays a critical role in protein assembly. In yeast, GRP78 knockout was found to cause a rapid block in protein secretion [73]. However, GRP78 has been shown to be upregulated by bacterial infection in the Atlantic salmon, as well as during macrophage development in the goldfish [74,75]. Upregulation of GRP78 has been observed in the liver of the grass carp (*Ctenopharyngodon idella*) under cold stress (4°C) [76]. In our study, two GRP78 protein spots with similar pI shift and matching identical protein accession number had reduced intensity upon exposure of milkfish to hypothermal stress compared to that in untreated controls. The higher molecular weight observed for the GRP78 2D protein spot detected along the horizontal direction of the gel might be due to post-translational modifications such as mono-ADP-ribosylation or phosphorylation, which previous studies have shown to be essential for GRP78 activation under cold stress [77]. Moreover, we have observed higher EF-G levels in the gill of milkfish exposed to nonlethal low-temperature in comparison with controls, indicating increased sensitivity to hypothermal environments, along with higher protein biosynthesis rate. Significant changes in protein abundance were also seen for AlaRS, an important biomarker of low temperature acclimation that were increased in the gill of milkfish exposed to nonlethal low-temperature, and GRP75, a critical oxidative stress biomarker which we found increased in the gill of milkfish exposed to lethal hypothermal environment.

### Energy production in the gill

Two of the proteins identified in milkfish gill were classified as energy production markers: AR and ATPB5. AR is the first and rate-limiting enzyme in the polyol pathway of glucose metabolism, and catalyzes the reduction of glucose to sorbitol as well as the reduction of glutathione conjugates of unsaturated aldehydes. Recent studies have suggested that the induced activation of the polyol pathway could affect the NADPH/NADP ratio, influence the glutathione reductase/peroxidase system, as well as decrease the ratio of reduced glutathione to oxidized glutathione (GSH/GSSG), thereby causing oxidative stress. AR also plays a pivotal role as a mediator of cytokines, growth factors, and LPS-induced inflammation. [78,79]. ATP5B is a subunit of ATP synthase that catalyzes the rate-limiting step of ATP formation, and can utilize proton gradient to drive ATP synthesis in the inner mitochondrial membrane. Proteomics analysis of melanoma cells exposed to H_2_O_2_-induced oxidative stress demonstrated that ROS increase ATP5B expression levels, and that inhibition of ATP5B activity affects apoptotic cell death [80]. Our study showed that lethal low-temperature increased ATP5B levels, suggesting a role for ATP5B in maintaining energy production under oxidative stress environment in order to preserve homeostasis in milkfish gill.

### Intracellular homeostasis in the gill

Two gill proteins identified upon exposure of milkfish to lethal low-temperature were classified into the compensatory response category. These proteins were CaM and CAIII. CaM is a multifunctional calcium-binding messenger protein in calcium-mediated signal transduction. Antarctic notothenioid fish (*Dissostichus mawsoni*) dwelling in seawater at temperatures between -2°C and 4°C have shown very high expression levels of CaM. Overexpression of the CaM gene from icefish increased cold tolerance in tobacco because of the CaM-mediated inhibitory effect on lipid peroxidation [81]. CaM gene expression might also play a role in immune response against WSSV and bacterial infection in crab and shrimp. CaM-mediated immune response leads to the activation of targets genes such as IL-6 and IL12b, which are involved in B cells inflammation and maturation in the Chinese mitten crab [82]. Environmental pH stress significantly alters CaM expression levels in gill, hepatopancreas, and muscle tissue of the Chinese mitten crab [82]. Moreover, the effects of hypothermia on epithelial Ca^2+^ channel gene expression and Ca^2+^ influx have been associated with pH imbalance in zebrafish gill [30]. CA is a zinc metalloenzyme, which is likely to contribute to branchial CO_2_ excretion and ion transport. In the gills, cytosolic carbonic anhydrase (CAc) catalyzes the hydration of CO_2_ to HCO_3_^-^, thereby exerting CO_2_ excretion ionic regulation and acid-base regulation functions. In the rainbow trout, branchial CAc mRNA expression, protein levels, and activity were significantly changed by acid and base infusion, suggesting that CAc expression is involved in compensatory responses to altered acid-base homeostasis [83]. CAc expression has also been investigated in the aquatic air-breathing fish, which has the ability to exchange gases through a labyrinth organ (LO). Although CAc is an important enzyme for gas exchange and acid-base balance, no significant change has been detected in the amounts of CAc in the gills and LO of the aquatic air-breathing fish under hypoxia stress [84]. In our study, increased levels of CaM in milkfish exposed to lethal hypothermal environment indicate the involvement of CaM in maintaining pH homeostasis, suggesting that this protein could be a biomarker of pH imbalance.

## Conclusion

In this proteomics study, we have identified important protein markers of physiological and metabolic response to lethal and nonlethal hypothermal stress in milkfish liver and gills. These proteins perform different physiological and metabolic functions at low temperature and their abundance differed between liver and gill. When exposed to nonlethal low-temperature, oxidative stress in the liver led to increased levels of antioxidant proteins. However, proteins related to inflammation response and protein quality control would be recruited in the gill. Upon lethal hypothermal environment, calcium concentration imbalance led to apoptosis in milkfish liver and altered levels of critical protein markers suggested a compensatory response to regulate calcium and bicarbonate homeostasis. These findings will provide a better understanding of putative compensatory response mechanisms to hypothermal stress in milkfish during the cold snap in winter.
